# Platelet Activation and Inflammation in Patients with Papillary Thyroid Cancer

**DOI:** 10.3390/diagnostics11111959

**Published:** 2021-10-22

**Authors:** Sorina Martin, Theodor Mustata, Oana Enache, Oana Ion, Andreea Chifulescu, Anca Sirbu, Carmen Barbu, Adrian Miron, Cosmin Giulea, Florin Andrei, Simona Fica

**Affiliations:** 1 Endocrinology Department, Carol Davila University of Medicine and Pharmacy, 020021 Bucharest, Romania; sorina.martin@gmail.com (S.M.); ancaelenasirbu@yahoo.com (A.S.); carmen_gabriela_barbu@yahoo.co.uk (C.B.); simonafica55@gmail.com (S.F.); 2 Endocrinology Department, Elias Hospital, 011461 Bucharest, Romania; oana.budianu@yahoo.ro; 3 Nephrology Department, Fundeni Clinical Institute, 022328 Bucharest, Romania; oana.catalina_ion@yahoo.ro; 4 Gastroenterology Department, Fundeni Clinical Institute, 022328 Bucharest, Romania; andreea.grigore92@yahoo.com; 5 Surgery Department, Carol Davila University of Medicine and Pharmacy, 020021 Bucharest, Romania; dramiron@yahoo.com; 6 Surgery Department, Elias Hospital, 011461 Bucharest, Romania; gcosmin@yahoo.com; 7 Pathology Department, Elias Hospital, 011461 Bucharest, Romania; florina6@yahoo.com

**Keywords:** differentiated thyroid cancer, papillary thyroid cancer, inflammatory markers, platelet indices

## Abstract

Background: The primary endpoint was to analyze the preoperatory inflammatory markers and platelet indices in papillary thyroid cancer (PTC) patients compared with patients with benign thyroid pathology. The secondary endpoints were to analyze the relationship between these markers and the pathological features of PTC and to compare their pre- and postoperative levels in PTC patients. Methods: In this retrospective case-control study, we analyzed the files of 1183 patients submitted to thyroidectomy between January 2012 and December 2018. A total of 234 patients with PTC (mean age 51.54 ± 13.10 years, 84.6% females) were compared with an age-, gender- and BMI-matched control group of 108 patients with histologic benign thyroid disorders. Results: PTC patients had higher platelet count (PLT) (*p* = 0.011), plateletcrit (PCT) (*p* = 0.006), neutrophil (*p* = 0.022) and fibrinogen (*p* = 0.005) levels. Subgroup analysis showed that PTC females had higher PLT (*p* = 0.006), PCT (*p* < 0.001) and erythrocyte sedimentation rate (ESR) (*p* = 0.005), while males had higher neutrophil (*p* = 0.040) levels. Papillary thyroid cancer patients under 55 years had higher PLT (*p* < 0.001) and PCT (*p* = 0.010), while patients over 55 years had higher mean platelet volume (*p* = 0.032), neutrophil-to-lymphocyte ratio (*p* = 0.013), ESR (*p* = 0.005) and fibrinogen (*p* = 0.019) levels. Preoperative values for platelet indices and inflammatory markers were similar to the postoperative determinations in PTC patients. Fibrinogen (AUROC = 0.602, *p* = 0.02; cut-off = 327.5 mg/dL, Se = 53.8%, Sp = 62.9%) and PLT (AUROC = 0.584, *p* = 0.012; cut-off = 223.5 × 10^3^/mm^3^, Se = 73.1%, Sp = 42.6%) were independent predictors of the presence of PTC. Conclusions: Our data show that fibrinogen and platelet count could be promising, inexpensive, independent predictors for the presence of PTC when compared with benign thyroid disorders.

## 1. Introduction

Thyroid carcinomas, the most common type of endocrine malignancies, are 2–4 times more common in women than men, and their incidence has rapidly increased during the past decades, mostly due to increased incidence of papillary thyroid carcinomas (PTCs) [1]. The prevalence of thyroid nodules ranges from 5% by palpation to 19–68% by high resolution ultrasonography [2]. Ultrasonography and fine-needle aspiration biopsy play important roles in the diagnosis and follow-up of thyroid nodules. In addition, a variety of other parameters have been investigated in order to differentiate between benign and malignant thyroid nodules and to select appropriate treatments.

Although the relationship between cancer and inflammation has been known since 1863 when Rudolf Virchow hypothesized that chronic inflammation could contribute to the tumorigenic process, increasing attention has been recently focused on inflammation and immunity as major mechanisms involved in thyroid tumorigenesis [3]. Furthermore, chronic inflammation plays important roles both in tumor progression as well as in immune surveillance and responses to therapy. The possible mechanisms by which inflammation can contribute to carcinogenesis include induction of genomic instability, alterations in epigenetic events and subsequent inappropriate gene expression, enhanced proliferation of initiated cells, resistance to apoptosis, aggressive tumor neovascularization, invasion through tumor-associated basement membrane and metastasis [4,5,6]. Conversely, the tumor itself promotes an inflammatory reaction by secretion of proinflammatory factors or by development of tumor necrosis [7,8].

The relationship between platelets (PLT) and cancer is not fully understood. Nonetheless, recent studies show that platelets and malignant cells influence one another. Malignant cells stimulate platelets production (paraneoplastic thrombocytosis) and cause platelets activation and aggregation. Conversely, platelets sustain tumor growth, tissue invasion and metastases [9].

Neutrophil count, lymphocyte count, the neutrophil-to-lymphocyte ratio (NLR), the lymphocyte-to-monocyte ratio (LMR), PLTs and platelet indices (platelet distribution width (PDW), plateletcrit (PCT) and mean platelet volume (MPV)), as indicators of platelet morphology and activation, could be used as inflammatory markers in cancer patients in addition to their use in cardiovascular, cerebrovascular, thromboembolic and inflammatory diseases [10]. Nevertheless, these potentially related carcinoma markers are inconsistent in PTC patients, with studies reporting discrepant results. Seretis et al. were one of the first groups to study the potential association of PTC and NLR and supposed that NLR may predict the presence of occult papillary thyroid microcarcinoma (PTMC) in otherwise benign goiters [11]. Gong et al. also reported that the preoperative NLR was closely related to the stage of papillary thyroid carcinoma [12]. NLR was also shown to be a prognostic factor for recurrence in low-risk DTC patients [13], and a correlation between NLR and tumor size in thyroid cancers has been shown by Cheong et al. [14]. Additionally, recent studies suggest that platelet indices may be possible markers in the differential diagnosis of benign and malignant thyroid disorders. MPV values were found to be significantly higher in patients with PTC compared with healthy patients or patients with nodular goiter [15]. There was also reported a significant decrease in MPV after thyroidectomy in patients with PTC, which was not observed in patients with nodular goiter [16]. Lower PDW and higher PCT values were observed in PTC patients when compared with healthy and benign thyroid disordered patients [17]. Li et al. recently suggested that the combined use of PDW and albumin might be useful in distinguishing thyroid cancer from benign thyroid nodules [18]. More than that, Wen et al. reported that pretreatment peripheral indexes, such as neutrophils, lymphocytes, MPV, PDW and LMR, could function as inexpensive indicators of aggressive behavior and higher stage in elderly patients with PTC [19]. However, there are studies that found no significant relationship between PTC and inflammatory hematological parameters including, in particular, NLR and MPV [20]. Machairas et al. found that white blood cell and platelet indices cannot assist in distinguishing benign goiter from thyroid cancer, but, in PTC patients, they can provide information about tumor multifocality and extrathyroidal extension [21]. Manatakis et al. also stated that NLR and platelet-to-lymphocyte ratios (PLR) cannot effectively predict the presence of occult papillary microcarcinomas in benign, multinodular goiters [22].

Therefore, the primary endpoint of the present retrospective study was to analyze the preoperatory inflammatory markers and platelet indices in PTC patients compared with patients with benign thyroid pathology. The secondary endpoints were to analyze the relationship between inflammatory markers and platelet indices and pathological features of PTC and to compare pre- and postoperative inflammatory markers and platelet indices in PTC patients.

## 2. Materials and Methods

### 2.1. Patients and Study Protocol

**Figure 1:**
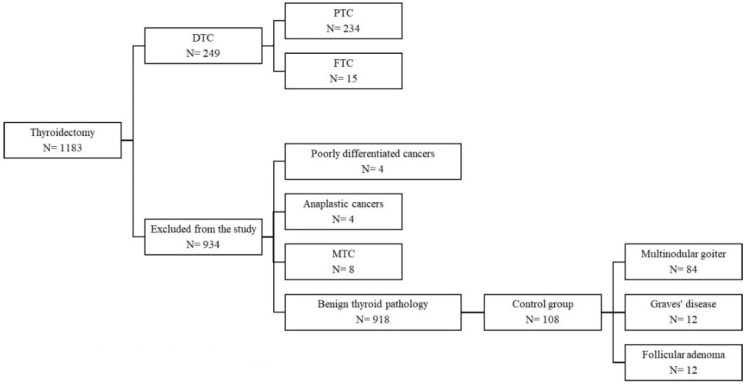
Flow diagram of the patient selection procedure.

We conducted a hospital-based retrospective case-control study using the data from 1183 files of patients who underwent total thyroidectomy or lobectomy in our surgery department between January 2012 and December 2018. A total of 265 (22.40%) of these patients were diagnosed with thyroid cancer. The study population included 249 (93.96%) patients with histologic differentiated thyroid cancer (DTC), 234 (88.3%) papillary thyroid carcinomas and 15 (5.66%) follicular thyroid carcinomas (FTC). Exclusion criteria were poorly differentiated thyroid cancers (4, 1.51%), anaplastic cancers (4, 1.51%), medullary thyroid carcinomas (MTC) (8, 3.02%) and patients with histologic benign thyroid pathology (918). The controls were selected from the same cohort of 1183 patients and included the first 108 age-, gender- and BMI-matched subjects with histologic benign thyroid pathology: multinodular goiter (84), follicular adenoma (12) and Graves’ disease (12) (Figure 1). Due to the retrospective nature of the study and the recent change in nomenclature that saw EFVPTC (encapsulated follicular variant of papillary thyroid carcinoma) redefined as NIFTP (noninvasive follicular thyroid neoplasm with papillary-like nuclear features) [23], we did not include patients diagnosed with NIFTP after 2016 and did not analyze patients based on the PTC histological subtype.

For all the 249 DTC patients, we recorded the following preoperative clinical and paraclinical data: age, gender, body mass index (BMI), fibrinogen, erythrocyte sedimentation rate (ESR), platelet count, MPV, PDW, PCT, TSH, FT4, anti- thyroid peroxidase antibodies (TPOAb), anti- thyroglobulin antibodies (ATA), anti-TSH receptor antibodies (TRAb) and pathological report data describing tumor features that included pathological variant of DTC–follicular and papillary carcinoma subtypes, cancer size, pTNM stage [21], multifocality, vascular invasion, capsular invasion, extracapsular extension, locoregional lymph node involvement and presence of thyroiditis. In addition, we calculated the neutrophil-to-lymphocyte (NLR) and platelet-to-lymphocyte (PLR) ratios as follows: NLR was calculated as the absolute neutrophil count divided by the absolute lymphocyte count, and PLR was calculated as the absolute platelet count divided by the absolute lymphocyte count, based on the preoperative blood count. For 63 PTC patients, the following postoperative data were also available to be recorded at a mean time of 3 months after surgery at the first follow-up visit: ESR, platelet count, MPV, PDW, PCT. For the 108 patients with benign thyroid pathology who were included in the control group, preoperative data were collected: age, gender, BMI, fibrinogen, ESR, platelet count, MPV, PDW, PCT, NLR, PLR and pathological report diagnosis.

Fasting baseline blood samples were routinely obtained the day before surgery and included hematocrit, hemoglobin, total WBC, automated differential counts (neutrophils, lymphocytes, monocytes, basophils and eosinophils) and platelets using a Sysmex XN-1000TM Hematology Analyzer (Sysmex Corporation, Kobe, Japan). Normal values for platelet indices were as follows: platelet count = 150–400 × 10^3^/mm^3^; MPV = 6.7–11.5 fL; PDW = 10.1–16.1%; PCT = 0.17–0.32%. Normal ESR = 0–20 mm/h and fibrinogen = 238–498 mg/dL. Serum TSH, FT4 and anti-thyroid antibodies were evaluated in a single laboratory by autoimmune amplified chemiluminescence immunochemistry using Immulite 2000 (Siemens Healthcare Diagnostics Products Ltd., Erlangen, Germany). Antithyroid antibody status was defined as follows: ATA positive = patients with ATA serum levels >40 IU/mL; TPOAb positive = patients with TPOAb serum levels >35 IU/mL; TRAb positive = patients with TRAb serum levels >1.5 IU/L. Autoimmune thyroiditis was diagnosed based on thyroid antibody positivity and/or the pathological diagnosis.

### 2.2. Data Presentation and Statistical Analyses

The distribution of the continuous quantitative variables was evaluated by the Shapiro–Wilk normality test. Descriptive data are presented as means ± SD, medians with interquartile range (IQR) or percentage. Between groups comparisons were carried out using parametric (independent sample *t*-test, *t*-test for pairwise samples, one-way analysis of variance (ANOVA) for more than two independent groups) or nonparametric (Mann–Whitney U-test, Kruskal–Wallis one-way ANOVA, Kolmogorov–Smirnov) tests, as appropriate. Chi-square test and Fisher’s exact test were used to compare proportions in large, respectively small groups. Relations between continuous variables were analyzed using Pearson’s correlation parametric coefficient or Spearman’s rho nonparametric correlation coefficient. Linear regression analyses and logistic regression analyses were used to identify the influence of inflammatory markers and platelet indices in PTC patients. Results are presented as odds ratio (OR) for the predictor variable, 95% confidence interval (CI) and *p* value for each variable assumed as a predictor. The overall validity of the model was measured using area under the receiver operating characteristic curve (AUROC) with 95% CI. The SPSS statistical package for Windows, version 20.0. (IBM Corp. Released 2011. IBM SPSS Statistics for Windows, Version 20.0. Armonk, NY, USA: IBM Corp.), was used to perform all statistical analysis. A *p* value < 0.05 indicated statistical significance.

## 3. Results

The clinical and laboratory characteristics of the study population consisting of 234 patients with PTC compared with 108 age-, gender- and BMI-matched control patients with histologic benign thyroid pathology are described in Table 1. PTC patients had significantly higher median levels of TSH (1.21 (1.38) vs. 0.87 (1.23) µIU/mL, *p* = 0.032), while FT4 median levels were similar in the two groups (*p* = 0.515). In our patient population, the prevalence of autoimmune thyroiditis was higher in the control group than in the PTC group (88.9% vs. 48.7%, χ^2^ = 50.315, *p* < 0.001). The pathological reports data in our PTC patients displayed multifocality in 31.2%, vascular invasion in 12.8%, capsular invasion in 32.1%, extracapsular extension in 20.9% and lymph node metastases in 15.8% of cases. Median tumor size was 10 (17) mm and most patients had either T1 or T2 tumors (66.7%) according to the pTNM staging system [24].

Univariate comparative analysis of the inflammatory markers and platelet indices according to the type of DTC (Table 2) showed that patients with PTC had a significantly higher mean platelet count (263.82 ± 65.44 vs. 244.62 ± 61.59 × 10^3^/mm^3^, *p* = 0.011) and PCT levels (0.28 ± 0.06 vs. 0.26 ± 0.06%, *p* = 0.006) but similar mean MPV (0.846) and median PDW (*p* = 0.967) levels when compared with controls. Significantly higher median neutrophil count levels were observed in the PTC group when compared with the control group (4.44 (1.91) vs. 4.05 (1.83) × 10^3^/mm^3^, *p* = 0.022). Median lymphocyte count (*p* = 0.976), NLR (*p* = 0.201) and PLR (*p* = 0.727) levels were not statistically different compared with controls. Median ESR values did not differ significantly, but patients with both DTC types had significantly higher mean levels of fibrinogen than those in the control group (338.29 ± 70.64 vs. 307.9 ± 68.01 mg/dL, *p* = 0.005 in PTC group, 357 ± 62.59 vs. 307.9 ± 68.01 mg/dL, *p* = 0.029 in FTC group, respectively).

Univariate comparative analysis of the inflammatory markers and platelet indices according to gender revealed that females had significantly higher PLT (268.84 ± 65.11 vs. 236.19 ± 60.95 × 10^3^/mm^3^, *p* = 0.006), PCT (0.29 ± 0.06 vs. 0.24 ± 0.05%, *p* < 0.001) and ESR (13 (12) vs. 6 (9) mm/h, *p* = 0.005), while males had a higher neutrophil count (4.82 (2.11) vs. 4.37 (1.87) × 10^3^/mm^3^, *p* = 0.040) (Table 3).

Lining up to the age cutoff chosen by the new TNM classification [24], we divided patients with PTC into two age groups: under and over 55 years old. Univariate comparative analysis of the inflammatory markers and platelet indices according to the age group showed that patients under 55 years old had significantly higher PLT (278.96 ± 62.84 vs. 246.75 ± 64.37 × 10^3^/mm^3^, *p* < 0.001) and PCT (0.29 ± 0.05 vs. 0.27 ± 0.06%, *p* = 0.010), while patients over 55 years old had higher MPV (10.85 ± 1.11 vs. 10.56 ± 0.94 fL, *p* = 0.032), NLR (2.43 (1.29) vs. 2.06 (1.13), *p* = 0.013), ESR (16 (13) vs. 9 (11) mm/h, *p* = 0.005) and fibrinogen levels (352.43 ± 74.05 vs. 325.09 ± 65.05 mg/dL, *p* = 0.019) (Table 3).

Univariate comparative analysis of the inflammatory markers and platelet indices according to weight categories in PTC patients did not disclose any significant differences (data not shown).

Pathological report characteristics displayed that PTC patients with lymph node metastases had lower mean fibrinogen levels (314.98 ± 68.01 vs. 344.11 ± 70.36 mg/dL, *p* = 0.047) and were younger (47.3 ± 12.91 vs. 52.34 ± 13.01 years, *p* = 0.031) than those without lymph node metastases. Furthermore, patients with pathological autoimmune thyroiditis were older (53.6 ± 11.26 vs. 50.18 ± 14.06 years, *p* = 0.041), had higher mean MPV (10.95 ± 0.96 vs. 10.53 ± 1.04 fL, *p* = 0.002) and median PDW (13.3 (2.9) vs. 12 (3.4)%, *p* = 0.009) values compared with patients without autoimmune thyroiditis. Platelet indices and inflammatory markers were not influenced by the presence of vascular invasion, capsular invasion, extracapsular extension, multifocality or pT classification (Table 4, Table 5 and Table 6). Patients with T3 and T4 tumors were significantly older than those with T1 and T2 tumors (54.06 ± 12.94 vs. 50.28 ± 13.04 years, *p* = 0.037).

Overall, preoperative values for platelet indices (platelets count, MPV, PCT, PDW) ESR, neutrophil count, lymphocyte count, NLR and PLR were similar to the postoperative determinations in PTC patients (Table 7). However, when analyzing the PTC patients based on the pathological report findings (multifocality, capsular invasion, vascular invasion, extracapsular extension, lymph nodes metastasis, autoimmune thyroiditis, pT stage; data not shown) our data exhibit lower median ESR levels before thyroidectomy in patients with no capsular invasion (12 (10) vs. 14 (14) mm/h, *p* = 0.043), and no extracapsular extension, respectively (12 (13) vs. 14 (14) mm/h, *p* = 0.030). When it comes to platelet indices, we found lower mean PLT before surgery in PTC patients with vascular invasion (233.63 ± 62.06 vs. 262.13 ± 55.97 × 10^3^/mm^3^, *p* = 0.013).

Simple linear regression analysis showed that, in PTC patients, higher platelet-to-lymphocyte ratio is associated with larger cancer diameter (coefficient = 0.042, 95% CI = 0.002–0.083, *p* = 0.042).

**Figure 2:**
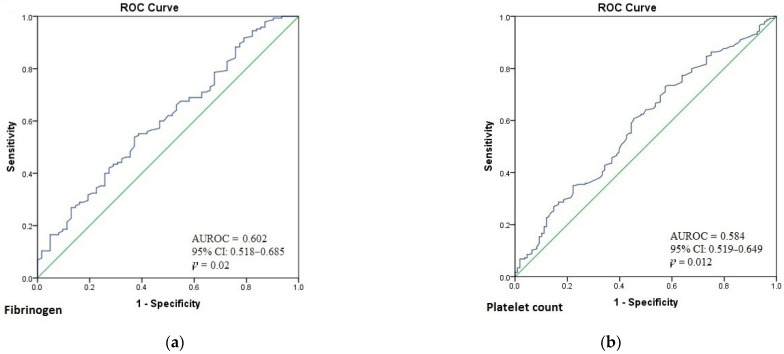
(**a**) Receiver operating characteristic (ROC) curve for the predictive value of fibrinogen on the presence of PTC; (**b**) Receiver operating characteristic (ROC) curve for the predictive value of platelet count on the presence of PTC.

Multivariate binary logistic regression analysis was performed to ascertain the effects of preoperative platelet indices and inflammatory markers on the likelihood that the patients have PTC. The logistic regression model was statistically significant, χ^2^(6) = 13.559, *p* = 0.035. The model explained 10.5% (Nagelkerke R2) of the variance in PTC and correctly classified 67.8% of cases. Fibrinogen (OR = 1.007, 95% CI = 1.001–1.013, *p* = 0.026) and platelet count (OR = 1.008, 95% CI = 1.001–1.014, *p* = 0.018) were independent predictors of the presence of PTC. There was a good capacity of fibrinogen (AUROC = 0.602, 95% CI: 0.518–0.685, *p* = 0.02; cut-off = 327.5 mg/dL, Se = 53.8%, Sp = 62.9%) (Figure 2a) and platelet count (AUROC = 0.584, 95% CI: 0.519–0.649, *p* = 0.012; cut-off = 223.5 × 10^3^/mm^3^, Se = 73.1%, Sp = 42.6%) (Figure 2b) to predict the presence of PTC.

## 4. Discussion

In this retrospective study we analyzed the preoperatory inflammatory markers and platelet indices in 234 PTC patients compared with an age, gender and BMI control group of 108 patients with benign thyroid pathology. Furthermore, we investigated the relationship between inflammatory markers and platelet indices and pathological features of PTC and we compared pre- and postoperative inflammatory markers and platelet indices in PTC patients.

Xenograft experiments and transgenic mouse models demonstrated that platelet activation and platelet–cancer cell interaction are crucial for cancer development and metastasis. Growth factors, metabolites and microRNA released by activated platelets induce epithelial-to-mesenchymal transition and enhance cancer cell stemness, which is crucial for cancer cell colonization at the distant organs. Direct or indirect interaction of platelets induces cancer cell plasticity and enhances survival and extravasation of circulating cancer cells during dissemination. On the other hand, in vivo and in vitro experiments demonstrate that cancer cells induce platelet activation and aggregation, subsequently elevating the risk of thrombosis, suggesting that platelet–cancer interaction is bidirectional [9]. Furthermore, platelets interactions with cancer cells and the tumor microenvironment are very complex and seem to have dual behaviors: pro and anti-cancerous, with the procancerogenic effect out-numbering the anti-cancerous effects [25]. Targeting platelet–cancer cell interaction may be a potential strategy for reducing both cancer metastasis and cancer-associated thrombosis [9].

Given their broad accessibility, low cost and high reproducibility rates, platelet count and their indices—MPV, PDW and PCT—have been used as inflammatory markers in cancer patients and the subject of numerous studies in different types of cancer, but there are scarce and conflicting data surrounding their role as biomarkers in thyroid cancer, possibly due to the small size population in the previous studies and the excellent prognosis and high survival rates.

Our PTC patients had higher PLT and PCT when compared with controls, a finding that can be explained by the previously described overproduction of interleukin-6 in malignancies, which stimulates the production of thrombopoietin and leads to megakaryocyte proliferation [26]. To the best of our knowledge, we are the first to show that PLT and fibrinogen are independent predictors for the presence of PTC. Using ROC analysis, we found a cutoff value of 327.5 mg/dL for fibrinogen to predict the presence of PTC with a sensitivity of 53.8% and a specificity of 62.9% and a cutoff value of 223.5 × 10^3^/mm^3^ for platelet number to predict the presence of PTC with a sensitivity of 73.1% and a specificity of 42.6%. Recently, Mizrak and Kucuk [27] also reported higher platelet count in PTC patients. Additionally, Dincel and Bayratkar noted similar results regarding PCT, but they found no significant difference in PLT between PTC and benign multinodular goiter patients [17]. Supporting our data, Jianyong et al. found that hyperfibrinogenemia is associated with advanced tumor stage and a high rate of recurrence, which enabled them to elaborate a nomogram that can be used to predict recurrence of papillary thyroid carcinoma [28].

Baldane et al. [16] reported that PTC patients have higher MPV values, which decrease after thyroidectomy, and their results were replicated by Ozmen et al. [29]. On the other hand, Yu et al. found lower MPV values in patients with thyroid cancer when compared with controls, but their study group included both DTC and MTC [30]. Our data showed no significant difference in MPV between PTC patients and controls. Additionally, we found no significant difference between preoperative and postoperative values for platelet indices and inflammatory markers, a finding that could be explained by the short time between surgery and the first follow-up visit, or by the connate inflammatory profile in cancer patients.

PDW also assesses the platelet volume, indicating their variation in size. Although often overlooked in everyday clinical practice, Xia et al. showed in their meta-analysis that a high PDW level is a prognostic factor for cancer and is associated with lymph node metastasis [10], while Dincel and Bayraktar obtained significantly lower PDW values in PTC patients when compared with controls [17]. Nevertheless, we could not find any significant difference in PDW levels among our study groups.

NLR and PLR have been widely studied as biomarkers in inflammatory conditions and cancer, but with inconsistent results in thyroid malignancies. A high number of neutrophils suppresses the immune system by inhibiting lymphocytes, activated T cells and natural killer cells in their cytolytic activity. On the other hand, lymphocytes and increased tumoral lymphocytic infiltration have been associated with better outcomes in cancer patients [31]. Seretis et al. were the first to show that NLR may predict the presence of PTC [11]. Subsequent studies showed higher NLR in PTC patients [22,32] and association with tumor size [14,33], extrathyroidal extension [33], lymph node metastases [34] or cancer recurrence [13]. Furthermore, a recent meta-analysis published by Feng et al. underlines the role of NLR as a biomarker for the prediction of tumor growth, metastasis and prognosis in patients with thyroid cancer [35]. Even though we did find a higher neutrophil count in the PTC group, we were not able to replicate the results of the previously mentioned studies. Our data did not reveal PLR as a useful marker in differentiating between PTC and benign thyroid disorders. However, consistent with the findings of Kim et al., who reported higher PLR in patients with tumors larger than 1 cm, in our PTC patients, higher PLR was associated with larger tumor size [36]. Looking further at the pathological reports and trying to find whether platelet indices or inflammatory markers can predict an aggressive tumor behavior, we surprisingly found lower fibrinogen levels in PTC patients with lymph node metastases—data that contradicts recent research on esophageal and gastric cancers [37,38]. However, we were able to replicate the results obtained by Wen et al. [19] regarding PDW and MPV, which were higher in patients with pathological autoimmune thyroiditis. Even though the presence of autoimmune thyroiditis was significantly higher in the control group, it was still highly prevalent in the PTC group as well, with almost half of the patients diagnosed with autoimmune thyroiditis based on thyroid antibody positivity and/or the pathological diagnosis. Published data show that up to one-third of PTC cases are associated with autoimmune thyroiditis [39]. For this reason, we chose to include all patients in the study, regardless of the presence of thyroid autoimmunity, in order to replicate the PTC cases that we evaluate in the clinical practice. While Machairas et al. [21] showed higher MPV in multifocal PTC and higher PLT, PCT and PLR in cases of extrathyroidal extension, platelet indices and inflammation did not differ depending on the presence of vascular invasion, capsular invasion, extracapsular extension, multifocality or pT classification in our study.

The strengths of this study are the important sample size of our thyroid cancer patients, the well-matched controls and the availability of pre- and postoperative data, as well as the detailed pathological reports. The main limitations are the retrospective nature of the study, which made it difficult to exclude other major factors that could have influenced fibrinogen levels and platelet indices, such as diabetes, smoking, infections, inflammatory diseases, iron deficiency and the use of antiplatelet drugs [40,41,42] and the lack of other inflammatory markers such as CRP (C-reactive protein), TNF-α (tumor necrosis factor-α), interleukin-6. The relationship between platelet indices, inflammatory markers and prognosis in PTC was not the subject of this paper, but it will be analyzed in a subsequent study as we gather more follow-up data.

## 5. Conclusions

Thyroid carcinomas are the most common type of endocrine malignancies, and their incidence has increased in the last decades due to widespread use of high-resolution ultrasonography. In the attempt to find markers that differentiate between benign and malignant thyroid nodules and help select appropriate treatments, our data show that fibrinogen and platelet count could be promising, inexpensive, independent predictors for the presence of PTC when compared with benign thyroid disorders.

## Figures and Tables

**Table 1:** Clinical and laboratory characteristics of the study population.

Parameter	Control Group N = 108	PTC N = 234	*p*
Age, mean ± SD (years)	52.07 ± 12.9	51.54 ± 13.10	0.726
Sex, N (%)			
Female	90 (83.3)	198 (84.6)	0.762
BMI, mean ± SD (kg/m^2^)	28.45 ± 5.79	28.79 ± 5.7	0.730
TSH, median (IQR) (µIU/mL)	0.87 (1.23)	1.21 (1.38)	**0.032**
FT4, median (IQR) (ng/dL)	1.14 (0.37)	1.08 (0.32)	0.515
Autoimmune thyroiditis, N (%)	96 (88.9)	114 (48.7)	**<0.001**

Abbreviations: BMI, body mass index; FT4, free T4; IQR, interquartile range; PTC, papillary thyroid carcinoma; SD, standard deviation; TSH, thyroid stimulating hormone.

**Table 2:** Univariate comparative analysis of the inflammatory markers and platelet indices according to the type of DTC.

Parameter	Control Group N = 108	DTC N = 249	*p* *	PTC N = 234	*p* +	FTC N= 15	*p* #	*p* ±
PLT, mean ± SD (×10^3^/mm^3^)	244.62 ± 61.59	263.12 ± 65.64	**0.013**	263.82 ± 65.44	**0.011**	252.2 ± 70.2	0.661	0.507
PCT, mean ± SD (%)	0.26 ± 0.06	0.28 ± 0.06	**0.007**	0.28 ± 0.06	**0.006**	0.27 ± 0.07	0.444	0.783
MPV, mean ± SD (fL)	10.68 ± 0.84	10.7 ± 1.02	0.821	10.7 ± 1.03	0.846	10.75 ± 0.83	0.762	0.855
PDW, median (IQR) (%)	12.4 (2.8)	12.4 (3.1)	0.997	12.45 (3.1)	0.967	11.9 (1.7)	0.925	0.987
Neutrophil count, median (IQR) (×10^3^/mm^3^)	4.05 (1.83)	4.42 (1.89)	**0.031**	4.44 (1.91)	**0.022**	4.09 (1.81)	0.920	0.309
Lymphocyte count, median (IQR) (×10^3^/mm^3^)	1.93 (0.8)	1.94 (0.7)	0.992	1.94 (0.7)	0.976	2.09 (0.66)	0.973	0.987
NLR, median (IQR)	2.04 (1.11)	2.13 (1.26)	0.218	2.13 (1.27)	0.201	2.07 (1.16)	0.973	0.987
PLR, median (IQR)	126.07 (49.68)	128.86 (61.42)	0.936	130.58 (61.56)	0.727	116.29 (55.59)	0.285	0.294
ESR, median (IQR) (mm/h)	13 (11)	13 (13)	0.743	13 (12)	0.802	12 (12)	0.759	0.857
Fibrinogen, mean ± SD (mg/dL)	307.9 ± 68.01	339.61 ± 70.08	**0.003**	338.29 ± 70.64	**0.005**	357 ± 62.59	**0.029**	0.395

Abbreviations: DTC, differentiated thyroid cancer; ESR, erythrocyte sedimentation rate; FTC, follicular thyroid carcinoma; IQR, interquartile range; MPV, mean platelet volume; NLR, neutrophil-to-lymphocyte ratio; PCT, plateletcrit; PDW, platelet distribution width; PLR, platelet-to-lymphocyte ratio; PLT, platelet count; PTC, papillary thyroid carcinoma; SD, standard deviation. *p* * value comparing DTC patients with controls; *p* + value comparing PTC patients with controls; *p* # value comparing FTC patients with controls; *p* ± value comparing FTC with PTC patients. Put in bold the *p* values that show statistical significance (*p* < 0.05).

**Table 3:** Univariate comparative analysis of the inflammatory markers and platelet indices according to gender and age in PTC patients.

Parameter	Gender			Age		
	Female N = 198	Male N = 36	*p*	<55 Years N = 124	>55 Years N = 110	*p*
PLT, mean ± SD (×10^3^/mm^3^)	268.84 ± 65.11	236.19 ± 60.95	**0.006**	278.96 ± 62.84	246.75 ± 64.37	**<0.001**
PCT, mean ± SD (%)	0.29 ± 0.06	0.24 ± 0.05	**<0.001**	0.29 ± 0.05	0.27 ± 0.06	**0.010**
MPV, mean ± SD (fL)	10.73 ± 1.01	10.52 ± 1.12	0.253	10.56 ± 0.94	10.85 ± 1.11	**0.032**
PDW, median (IQR) (%)	12.6 (3.15)	12.2 (3.4)	0.696	12.25 (2.85)	13.1 (3.43)	0.199
Neutrophil count, median (IQR) (×10^3^/mm^3^)	4.37 (1.87)	4.82 (2.11)	**0.040**	4.27 (1.69)	4.86 (2.14)	0.068
Lymphocyte count, median (IQR) (×10^3^/mm^3^)	1.96 (0.69)	1.81 (1.05)	0.626	1.98 (0.67)	1.87 (0.74)	0.291
NLR, median (IQR)	2.12 (1.1)	2.81 (2.06)	0.587	2.06 (1.13)	2.43 (1.29)	**0.013**
PLR, median (IQR)	131.55 (56.99)	118.96 (93.92)	0.587	135.24 (64.51)	125.88 (62.16)	0.238
ESR, median (IQR) (mm/h)	13 (12)	6 (9)	**0.005**	9 (11)	16 (13)	**0.005**
Fibrinogen, mean ± SD (mg/dL)	338.25 ± 68.94	338.5 ± 82.49	0.988	325.09 ± 65.05	352.43 ± 74.05	**0.019**

Abbreviations: ESR, erythrocyte sedimentation rate; IQR, interquartile range; MPV, mean platelet volume; NLR, neutrophil-to-lymphocyte ratio; PCT, plateletcrit; PDW, platelet distribution width; PLR, platelet-to-lymphocyte ratio; PLT, platelet count; PTC, papillary thyroid carcinoma; SD, standard deviation. Put in bold the *p* values that show statistical significance (*p* < 0.05).

**Table 4:** Univariate comparative analysis of inflammatory markers and platelet indices according to pathological report characteristics in PTC patients.

Parameter	Vascular Invasion			Capsular Invasion			Extracapsular Extension		
	Present N = 30	Absent N = 204	*p*	Present N = 75	Absent N = 159	*p*	Present N = 49	Absent N = 185	*p*
Age—mean ± SD (years)	53.77 ± 16.08	51.22 ± 12.62	0.411	53.07 ± 13.65	50.82 ± 12.81	0.222	54.33 ± 12.77	50.81 ± 13.12	0.094
Sex N (%)									
Female	23 (76.7)	175 (85.8)	0.275	61 (81.3)	137 (86.2)	0.339	38 (77.6)	160 (86.5)	0.123
Male	7 (23.3)	29 (14.2)	0.275	14 (18.7)	22 (13.8)	0.339	11 (22.4)	25 (13.5)	0.123
BMI—mean ± SD (kg/m^2^)	26.89 ± 5.53	29.16 ± 5.69	0.147	28.67 ± 5.61	28.86 ± 5.8	0.871	28.84 ± 5.88	28.77 ± 5.68	0.961
PLT—mean ± SD (×10^3^/mm^3^)	263.8 ± 62.66	263.82 ± 65.99	0.999	268.75 ± 67.78	261.50 ± 64.39	0.430	273.9 ± 72.54	261.15 ± 63.37	0.226
MPV—mean ± SD (fL)	10.58 ± 1.08	10.71 ± 1.02	0.505	10.6 ± 1.1	10.74 ± 0.99	0.328	10.44 ± 1.03	10.77 ± 1.02	0.051
PCT—mean ± SD (%)	0.27 ± 0.05	0.28 ± 0.06	0.604	0.278 ± 0.059	0.28 ± 0.06	0.788	0.281 ± 0.07	0.279 ± 0.006	0.840
PDW—median (IQR) (%)	12.75 (2.53)	12.45 (3.20)	0.828	12.3 (3.13)	12.7 (3.2)	0.643	12 (3)	12.7 (3.1)	0.302
Neutrophil count—median (IQR) (×10^3^/mm^3^)	4.24 (2.38)	4.44 (1.82)	0.884	4.46 (2.13)	4.39 (1.9)	0.928	4.76 (2.17)	4.39 (1.84)	0.478
Lymphocyte count—median (IQR) (×10^3^/mm^3^)	1.78 (0.72)	1.97 (0.70)	0.087	1.84 (0.93)	1.97 (0.65)	0.190	1.85 (0.9)	1.97 (0.67)	0.223
NLR—median (IQR)	2.29 (2.21)	2.13 (1.14)	0.845	2.2 (1.84)	2.11 (1.03)	0.889	2.31 (1.86)	2.13 (1.07)	0.872
PLR—median (IQR)	159.46 (89.74)	128.42 (54.60)	0.328	131.47 (62.9)	127.98 (61.68)	0.889	142.39 (62.58)	127.59 (62.17)	0.335
ESR—median (IQR) (mm/h)	13 (21)	13 (12)	0.791	12.5 (15)	13 (12)	0.999	13 (17)	12.5 (12)	0.976
Fibrinogen—mean ± SD (mg/dL)	319.18 ± 73.35	341.34 ± 70.01	0.194	324.82 ± 69.79	344.95 ± 70.46	0.107	327.66 ± 67.71	341.3 ± 71.45	0.337

Abbreviations: BMI, body mass index; ESR, erythrocyte sedimentation rate; IQR, interquartile range; MPV, mean platelet volume; NLR, neutrophil-to-lymphocyte ratio; PCT, plateletcrit; PDW, platelet distribution width; PLR, platelet-to-lymphocyte ratio; PLT, platelet count; PTC, papillary thyroid carcinoma; SD, standard deviation.

**Table 5:** Univariate comparative analysis of inflammatory markers and platelet indices according to pathological report characteristics in PTC patients.

Parameter	Multifocality			Lymph Node Metastases			Autoimmune Thyroiditis (HP)		
	Present N = 73	Absent N = 161	*p*	Present N = 37	Absent N = 197	*p*	Present N = 93	Absent N = 141	*p*
Age—mean ± SD (years)	50.26 ± 14.17	52.12 ± 12.56	0.314	47.3 ± 12.91	52.34 ± 13.01	**0.031**	53.6 ± 11.26	50.18 ± 14.06	**0.041**
Sex N (%)									
Female	61 (83.6)	137 (85.1)	0.764	28 (75.7)	170 (86.3)	0.100	83 (89.2)	115 (81.6)	0.111
Male	12 (16.4)	24 (14.9)	0.764	9 (24.3)	27 (13.7)	0.100	10 (10.8)	26 (18.4)	0.111
BMI—mean ± SD (kg/m^2^)	29.27 ± 6.58	28.63 ± 5.41	0.630	29.31 ± 6.99	28.72 ± 5.54	0.739	27.82 ± 4.99	29.53 ± 6.13	0.139
PLT—mean ± SD (×10^3^/mm^3^)	261.97 ± 66.33	264.66 ± 65.22	0.772	270.49 ± 62.78	262.57 ± 66	0.501	254.47 ± 68.75	269.99 ± 62.65	0.076
MPV—mean ± SD (fL)	10.78 ± 1.1	10.66 ± 1	0.425	10.4 ± 1.06	10.75 ± 1.01	0.057	10.95 ± 0.96	10.53 ± 1.04	**0.002**
PCT—mean ± SD (%)	0.27 ± 0.055	0.28 ± 0.062	0.350	0.278 ± 0.057	0.28 ± 0.06	0.847	0.27 ± 0.064	0.28 ± 0.057	0.359
PDW—median (IQR) (%)	12.75 (3.55)	12.4 (2.97)	0.440	11.8 (2.9)	12.8 (3.1)	0.137	13.3 (2.9)	12 (3.4)	**0.009**
Neutrophil count—median (IQR) (×10^3^/mm^3^)	4.33 (1.62)	4.57 (2.04)	0.186	4.42 (2.12)	4.44 (1.86)	0.955	4.33 (1.97)	4.46 (1.9)	0.669
Lymphocyte count—median (IQR) (×10^3^/mm^3^)	1.97 (0.88)	1.93 (0.68)	0.711	1.84 (0.57)	1.97 (0.72)	0.083	1.96 (0.6)	1.91 (0.74)	0.915
NLR—median (IQR)	2.09 (1.09)	2.16 (1.28)	0.778	2.31 (1.97)	2.13 (1.12)	0.858	2.09 (1.25)	2.21 (1.28)	0.593
PLR—median (IQR)	130.27 (62.66)	131.63 (62.51)	0.888	142.39 (62.76)	127.59 (64.02)	0.282	125.56 (52.7)	135.05 (64.71)	0.182
ESR—median (IQR) (mm/h)	13 (12)	12.5 (14)	0.750	9 (9)	13 (14)	0.138	13 (14)	12 (12)	0.843
Fibrinogen—mean ± SD (mg/dL)	324.22 ± 63.58	345.46 ± 73.26	0.087	314.98 ± 68.01	344.11 ± 70.36	**0.047**	346.43 ± 68.12	332.7 ± 72.18	0.251

Abbreviations: BMI, body mass index; ESR, erythrocyte sedimentation rate; IQR, interquartile range; MPV, mean platelet volume; NLR, neutrophil-to-lymphocyte ratio; PCT, plateletcrit; PDW, platelet distribution width; PLR, platelet-to-lymphocyte ratio; PLT, platelet count; PTC, papillary thyroid carcinoma; SD, standard deviation. Put in bold the *p* values that show statistical significance (*p* < 0.05).

**Table 6:** Univariate comparative analysis of the inflammatory markers and platelet indices according to pathological pT classification.

Parameter	TNM Staging—pT Classification							
	T1 N = 128	T2 N = 28	T3 N = 73	T4 N = 5	*p*	T1–T2 N = 156	T3–T4 N = 78	*p*
Age—mean ± SD (years)	51.54 ± 11.95	44.29 ± 15.65	54.19 ± 12.78	52.2 ± 16.57	**0.008**	50.28 ± 13.04	54.06 ± 12.94	**0.037**
BMI—mean ± SD (kg/m^2^)	28.89 ± 5.61	27.59 ± 4.82	29.36 ± 6.13	24.08 ± 6.86	0.493	28.53 ± 5.34	29.12 ± 6.18	0.612
PLT, mean ± SD (×10^3^/mm^3^)	256.89 ± 63.72	274.25 ± 65.49	269.04 ± 67.51	309.8 ± 77.62	0.171	259.9 ± 63.78	271.65 ± 68.38	0.196
PCT, mean ± SD (%)	0.279 ± 0.061	0.287 ± 0.063	0.277 ± 0.058	0.31 ± 0.087	0.744	0.28 ± 0.061	0.276 ± 0.059	0.849
MPV, mean ± SD (fL)	10.81 ± 0.97	10.6 ± 1	10.58 ± 1.12	10.02 ± 1.09	0.174	10.78 ± 0.97	10.54 ± 1.12	0.100
PDW, median (IQR) (%)	12.8 (3.1)	12.3 (3.65)	12.2 (3.55)	13.4 (-)	0.491	12.7 (3.1)	12.2 (3.5)	0.539
Neutrophil count, median (IQR) (×10^3^/mm^3^)	4.48 (1.9)	4.2 (1.78)	4.46 (2.16)	5.04 (3.56)	0.319	4.43 (1.88)	4.51 (2.16)	0.817
Lymphocyte count, median (IQR) (×10^3^/mm^3^)	1.97 (0.68)	2.01 (0.72)	1.85 (0.81)	1.66 (1.53)	0.683	1.97 (0.68)	1.85 (0.88)	0.248
NLR, median (IQR)	2.16 (1.05)	1.86 (1.16)	2.13 (1.67)	2.98 (3.35)	0.149	2.11 (1.05)	2.17 (1.72)	0.890
PLR, median (IQR)	125.01 (55.25)	126.65 (85.74)	133.83 (49.73)	186.92 (107.21)	0.362	125.82 (62.7)	136.24 (56.35)	0.332
ESR, median (IQR) (mm/h)	13 (13)	11.5 (12)	13 (18)	7 (23)	0.439	12 (12)	13 (18)	0.952
Fibrinogen, mean ± SD (mg/dL)	345.35 ± 70.04	334.67 ± 64.53	329.87 ± 75.52	308.8 ± 67.95	0.518	343.54 ± 68.5	327.68 ± 74.37	0.204

Abbreviations: BMI, body mass index; ESR, erythrocyte sedimentation rate; IQR, interquartile range; MPV, mean platelet volume; NLR, neutrophil-to-lymphocyte ratio; PCT, plateletcrit; PDW, platelet distribution width; PLR, platelet-to-lymphocyte ratio; PLT, platelet count; SD, standard deviation.

**Table 7:** Univariate comparative analysis of pre- and postoperative levels of inflammatory markers and platelet indices in PTC patients.

Parameter	Preoperative N = 234	Postoperative N = 63	*p*
PLT, mean ± SD (×10^3^/mm^3^)	266.35 ± 60.86	265.14 ± 69.39	0.865
MPV, mean ± SD (fL)	10.57 ± 1	10.51 ± 1.04	0.382
PCT, mean ± SD (%)	0.277 ± 0.054	0.275 ± 0.062	0.841
PDW, median (IQR) (%)	12.4 (3.5)	12.5 (2.85)	0.963
ESR, median (IQR) (mm/h)	12 (14)	14 (14)	0.088
Neutrophil count, median (IQR) (×10^3^/mm^3^)	4.55 (1.92)	4.46 (2.37)	0.672
Lymphocyte count, median (IQR) (×10^3^/mm^3^)	1.85 (0.76)	1.83 (0.71)	0.354
NLR, median (IQR)	2.49 (1.33)	2.27 (1.28)	0.653
PLR, median (IQR)	138.93 (51.38)	143.48 (78.72)	0.536

Abbreviations: ESR, erythrocyte sedimentation rate; IQR, interquartile range; MPV, mean platelet volume; NLR, neutrophil-to-lymphocyte ratio; PCT, plateletcrit; PDW, platelet distribution width; PLR, platelet-to-lymphocyte ratio; PLT, platelet count; PTC, papillary thyroid carcinoma; SD, standard deviation.

## Data Availability

The data presented in this study are available on request from the corresponding author.
